# Gold Nanoplates for a Localized Surface Plasmon Resonance-Based Boric Acid Sensor

**DOI:** 10.3390/s17050947

**Published:** 2017-04-25

**Authors:** Marlia Morsin, Muhamad Mat Salleh, Akrajas Ali Umar, Mohd Zainizan Sahdan

**Affiliations:** 1 Microelectronics & Nanotechnology-Shamsuddin Research Centre (MiNT-SRC), Institute of Integrated Engineering (I2E), Universiti Tun Hussien Onn Malaysia, Batu Pahat, Johor 86400, Malaysia; zainizan@uthm.edu.my; 2 Department of Electronic Engineering, Faculty of Electronic and Electrical Engineering, Universiti Tun Hussien Onn Malaysia, Batu Pahat, Johor 86400, Malaysia; 3 Institute of Microengineering and Nanoelectronics (IMEN), Universiti Kebangsaan Malaysia, Bangi, Selangor 43600, Malaysia; mms@ukm.edu.my (M.M.S.); akrajas@ukm.edu.my (A.A.U.)

**Keywords:** localized surface plasmon resonance, plasmonic sensor, gold nanoparticles, gold nanoplates, boric acid

## Abstract

Localized surface plasmon resonance (LSPR) properties of metallic nanostructures, such as gold, are very sensitive to the dielectric environment of the material, which can simply be adjusted by changing its shape and size through modification of the synthesizing process. Thus, these unique properties are very promising, particularly for the detection of various types of chemicals, for example boric acid which is a non-permitted preservative employed in food preparations. For the sensing material, gold (Au) nanoplates with a variety of shapes, i.e., triangular, hexagonal, truncated pentagon and flat rod, were prepared using a seed-mediated growth method. The yield of Au nanoplates was estimated to be ca. 63% over all areas of the sensing material. The nanoplates produced two absorption bands, i.e., the transverse surface plasmon resonance (t-SPR) and the longitudinal surface plasmon resonance (l-SPR) at 545 nm and 710 nm, respectively. In the sensing study, these two bands were used to examine the response of gold nanoplates to the presence of boric acid in an aqueous environment. In a typical process, when the sample is immersed into an aqueous solution containing boric acid, these two bands may change their intensity and peak centers as a result of the interaction between the boric acid and the gold nanoplates. The changes in the intensities and peak positions of t-SPR and l-SPR linearly correlated with the change in the boric acid concentration in the solution.

## 1. Introduction

Surface plasmon is a collective oscillation of free electrons at the surface of a metal stimulated by the electric field of light. Typical metals that commonly demonstrate this plasmonic phenomenon are gold [1,2], silver [3,4], platinum [5] and palladium [6,7,8]. Surface plasmon is very sensitive and responsive to changes in the dielectric constant of the surrounding medium [9,10] making it potential for sensing applications [11,12]. Furthermore, surface plasmon is noted to be more unique when it is locally confined in a nanostructure which generates a localized surface plasmon resonance (LSPR) effect [1,3,4,5,6,7,8,9,10,11,12,13,14]. It promises enhanced sensitivity to even small changes in the properties of the surrounding medium, due to its localized nature that spreads over an enhanced field. In addition, LSPR is also strongly influenced by the size and shape of the nanostructure sensing materials [15], improving their sensitivity and selectivity in the sensing applications. 

Metal nanostructures, especially gold, have attracted the attention of many researchers because of their unique surface plasmon resonance (SPR) properties, high bio-compatibility, and high-reactivity. They have been widely used in surface-enhanced Raman scattering (SERS) [16,17], photoelectronic devices [18], catalysis [19], and biomedical applications [9,10]. In sensor applications, gold nanosphericals are widely used as sensing materials [20,21,22,23]. The single absorption band that is associated with the transverse SPR (t-SPR) is produced from the sample and is normally used as a key parameter in the detection of analytes, such as gaseous molecules. Since morphology influences the LSPR properties of metal nanostructures, the use of nanostructures with different morphologies, such as nanorods [21,24,25] and nanoplates [26,27,28], is expected could increase the sensitivity as well as expand the selectivity properties of such metal nanostructures. 

This paper reports the LSPR sensing properties of gold nanoplates in the presence of boric acid. Au nanoplates were grown on a quartz substrate surface using the seed-mediated growth method [27,28,29]. The implementation of Au nanoplates in an LSPR sensor gave an additional parameter, namely longitudinal SPR (l-SPR), to measure the sensitivity of the sensor other than transverse SPR (t-SPR). Commonly, the gold nanorods are employed as the sensing material to obtain two response peaks of an LSPR sensor [21,26]. Meanwhile, boric acid is a pesticide that is normally used to kill termites, wood decay fungi, plants and insects such as cockroaches [30]. However, boric acid has been misused in food processing as well as used as a preservative and an additive in various foods [31] such as noodles, seafood, dairy and meat products, especially by small-scale producers. Thus, the proposed LSPR sensor demonstrates an alternative approach for high-sensitivity detection of boric acid, compared to the conventional technique [32] that uses time-consuming analytical methods. 

## 2. Materials and Methods

### 2.1. Preparation of Gold Nanoplates

The sensing material of Au nanoplates was prepared using the seed-mediated growth method, as previously reported [33]. The preparation of Au nanoplates involves two main steps, which are the seeding and growth processes. The seeding process was done to attach the seeds onto the surface of the substrate. The substrate was immersed into a seeding solution consisting of 0.5 mL of 0.01 M HAuCl_4_ (Sigma Aldrich, St. Louis, MO, USA), 2 mL of 0.01 M trisodium citrate (Wako Pure Chemical Industries, Ltd., Osaka, Japan), 0.5 mL of 0.1 M iced-cold aqueous NaBH_4_ (Sigma Aldrich, St. Louis, MO, USA), and 18 mL deionized water (DI water) for 2 h at room temperature. This process was followed by 5 h of the growth process. In this process, the growth solution consisting of 0.5 mL of 0.01 M HAuCl_4_, 10 mL of 1 mM PVP (Sigma Aldrich, St. Louis, MO, USA), 8 mL of 0.1 M CTAB (Sigma Aldrich, St. Louis, MO, USA), 0.1 mL of 0.1 M ascorbic acid (Wako Pure Chemical Industries, Ltd., Osaka, Japan) and 2 mL DI water was prepared to immerse the substrate with the nanoseeds. The anneal processes were completed after each seeding and growth process. 

### 2.2. Optical Sensor System Setup

The prepared Au nanoplates were used as a sensing material to detect the presence of boric acid in the solution. A sensor setup was developed to evaluate the sensing properties of the Au nanoplates for the boric acid [33,34]. The setup consisted of a sensor chamber with two inlets and a drawer, a light source (LS-1 tungsten halogen lamp), a duplex fiber optical probe system, a USB-2000 Ocean Optics spectrometer (Ocean Optics, Dunedin, FL, USA) and a computer with OOIBase32 software (Ocean Optics, Dunedin, Florida, USA) as the spectrum analyzer tool. The Au nanoplates sample was placed on the drawer inside the sensor chamber. The light source beam was transmitted using one of the fiber arms directed towards Au nanoplates sample, and was subsequently reflected back. The reflected light was collected by the other fiber arm and transmitted to the spectrometer. The sensing sensitivity was based on the change in the optical absorbance of the Au nanoplates upon the presence of boric acid (purchased from R&M Chemicals, Selangor, Malaysia) in the solution.

## 3. Results and Discussion

### 3.1. Gold Nanoplates Characterization

The Au nanoplates formation on the quartz substrate was confirmed by X-ray diffraction (XRD) (D8 Advance) and field emission scanning electron microscopy (FESEM) analysis (Zeiss Supra 55VP). The XRD result [33] has been compared to the JCPDS-004-0784 file for bulk Au. An exceedingly high peak at 38.15° that can be indexed as the (111)-crystallographic planes of face-centered cubic (fcc) Au nanocrystals was observed in the spectrum. There was another peak with a lower intensity observed at 44.25°, which is related to (200) fcc lattice planes. Thus, the product was characterized by (111) facet [27], indicating that the plane prefers to orient in parallel to the surface of the substrate.

**Figure 1:**
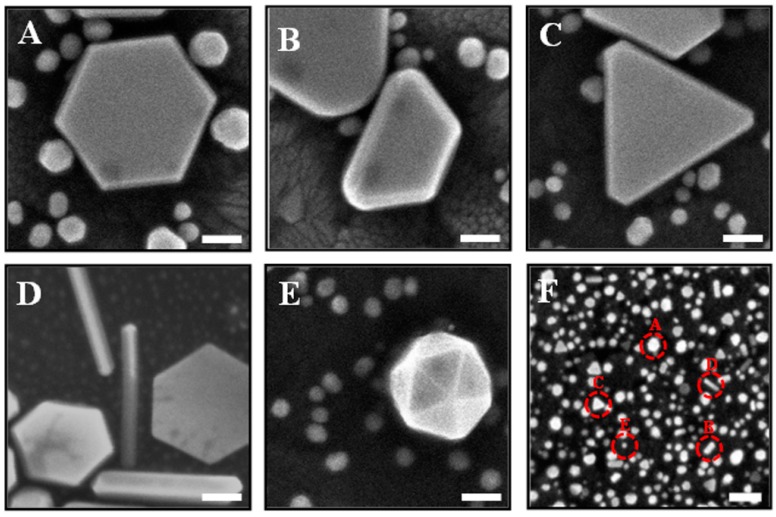
Field Emission Surface Scanning Electron Microscopy (FESEM) images of Au nanoplates grown on the substrate using the seed-mediated growth method. Variable structures of Au nanoplates were obtained, such as (**A**) symmetric hexagonal, (**B**) truncated pentagon, (**C**) triangular, (**D**) flat rod, (**E**) irregular shape; (**F**) the growth of Au in small sizes on the surface substrate. Scale bars are 100 nm.

The morphology of the Au nanoplates on the substrate was characterized using FESEM and is shown in Figure 1A–F. The figure shows that Au nanoplates with various shapes were grown on the substrate surface, such as triangular, truncated hexagonal, asymmetric hexagonal, symmetric hexagonal, truncated pentagon and flat rod. Moreover, spherical Au nanoparticles and irregular Au shapes were also observed. In this study, the growth time was fixed at 5 h. For the growth of Au nanoplates, plate formation started with a triangular shape and grew to a hexagonal shape. It was found that the hexagonal shapes make up the majority of the product. As seen from this figure, there are two groups of Au nanoplates with different sizes. The first group includes the Au nanoplates with an edge length of more than 150 nm, with a yield percentage of approximately 23% all over the surface area. The analysis was done by measuring the area covered by nanogold. Three different areas were measured and analyzed and the average of surface density was calculated. The second group includes the Au nanoplates with a smaller edge length (less than 50 nm), with a yield of up to ca. 40%. The bigger nanoplates are dominated by hexagonal shapes with an edge length of ca. 250 nm. The height of the Au nanoplates for both groups is between 10–30 nm. Moreover, the Au flat rod is also observed. The yield of the nanoplates can be estimated to be covering is about 63% of the surface area.

### 3.2. Plasmonic Sensing Response

**Figure 2:**
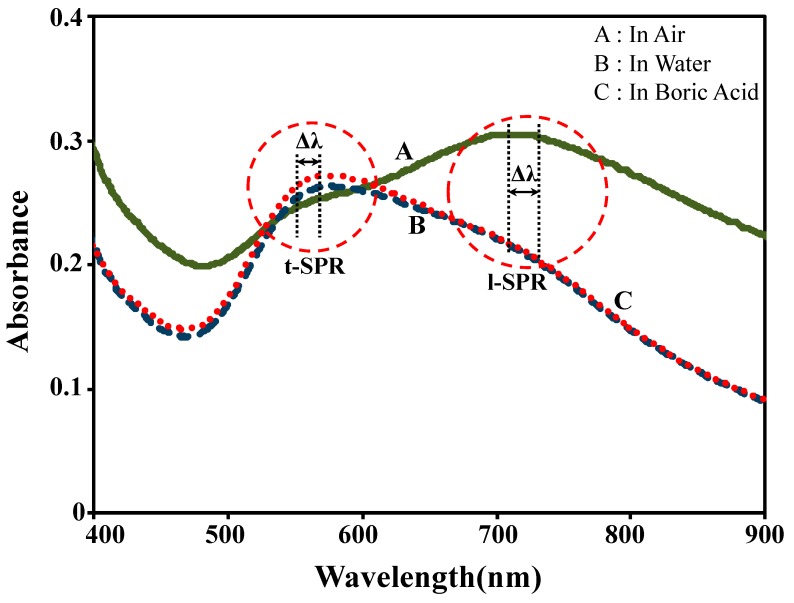
The spectra of Au nanoplates in three different mediums: (**A**) air, (**B**) deionized (DI) water, and (**C**) 1 mM boric acid.

The absorption spectra of Au nanoplates were studied in three mediums; air, DI water and 1 mM boric acid (H_3_BO_3_). The results for all spectra are shown in Figure 2. In the air medium, there are two absorption bands observed at 545 nm and 710 nm. The first peak is assigned as transverse SPR (t-SPR) and the second peak is assigned as longitudinal SPR (l-SPR) [19]. The t-SPR represents free charges vibration into the short axis (vertical direction), while l-SPR is the vibration of free charges into the longer axis (horizontal direction), which is parallel to the substrate surface. The t-SPR band agrees with previous observations of the spectrum of spherical shaped Au nanoparticles [21], and the l-SPR band agrees with the Au nanoplates spectrum [27,28]. Besides, it can be seen that the spectrum peak is broad and not very sharp due to the various shapes and sizes of Au nanoplates grown on the substrate. When the sample was immersed into the solution media, both DI water and boric acid (10 mM), two changes occurred in the spectrum that altered the peaks’ intensity and position. The peaks were increased at t-SPR, but attenuated at l-SPR when the medium was changed from air to solution. The presence of water and boric acid molecules in the surrounding medium influenced the resonance of the Au nanoplates samples. Instead of changes in intensity, it can be observed that the peak position tended to red-shifted with the change of medium. All these spectral changes can be described by the classical Mie theory [35], as shown in Equation (1):

(1) $$E\left(\lambda \right)=\frac{24\pi N_{A}a^{3}{\varepsilon_{m}}^{3/2}}{\lambda ln\left(10\right)}\left[\frac{\varepsilon_{i}}{{\left(\varepsilon_{r}+2\varepsilon_{m}\right)}^{2}+{\varepsilon_{i}}^{2}}\right]$$

where *|E*(*λ*)*|* is the extinction equal to the sum of absorption and Rayleigh scattering, *N_A_* is the area density of nanoparticles, *a* is the radius of the metallic nanosphere, *ε_m_* is the dielectric constant of the medium surrounding the metallic nanosphere, λ is the wavelength of the absorbing radiation, and *ε_i_* and *ε_r_* are the imaginary and the real portion of the metallic nanosphere dielectric function, respectively. The extinction coefficient depends on the nanoparticle’s in-plane diameter, out-of-plane height, and shape that can be shown by replacing the resonance term (*ε_r_* + 2*ε_m_*)^2^ with (*ε_r_* + *χε_m_*)^2^ where *χ* is a shape factor term that describes the nanoparticle’s aspect ratio. Meanwhile, for arbitrary shapes of small metal nanoparticles, Pennypacker and Purcell [35] presented a method called Discrete-Dipole Approximation (DDA) to compute scattering and absorption by particles. The DDA method is done by dividing nanoparticles to small particles as a set of small cubic subdensity which is also referred as bipolar. The dipole size must be smaller than the wavelength of the electromagnetic wave. The dipoles will interact with each other and the incident field. In this method, the response to light is measured by calculating the response of a dipole at the center of each cube to the absorbed and scattered light. The effects of the reaction depend on the size, shape and cubic dimensions. The improvement of this method has been continued by Draine et al. [36,37].

The change of the refractive index of the medium [38,39,40] can be measured using the following equation:

Δ*λ_max_* = *m*Δ*n*(1 − *exp*(−2*d/l_d_*)) (2)

where Δ*λ_max_* is the wavelength shift, *m* is the refractive index sensitivity, Δ*n* is the change in refractive index induced by an adsorbate, *d* is the effective adsorbate layer thickness, and *l_d_* is the characteristic electromagnetic field decay length.

To analyze the sensitivity of Au nanoplates, the concentration of boric acid was varied from 0.01 mM (0.614 mg/L) to 200 mM (12 368 mg/L). The Arago-Biot equation [41] as shown below, was used to determine the refractive index for each boric acid concentration:

*n*_12_ = *n*_1_*ϕ*_1_ + *n*_2_*ϕ*_2_ (3)

where *n*_12_ is the refractive index of the liquids mixture, *n*_1_ is the refractive index of the first liquid, *n*_2_ is the refractive index of the second liquid, *ϕ*_1_ is the mole fraction of the first liquid and *ϕ*_2_ is the mole fraction of the second liquid.

**Figure 3:**
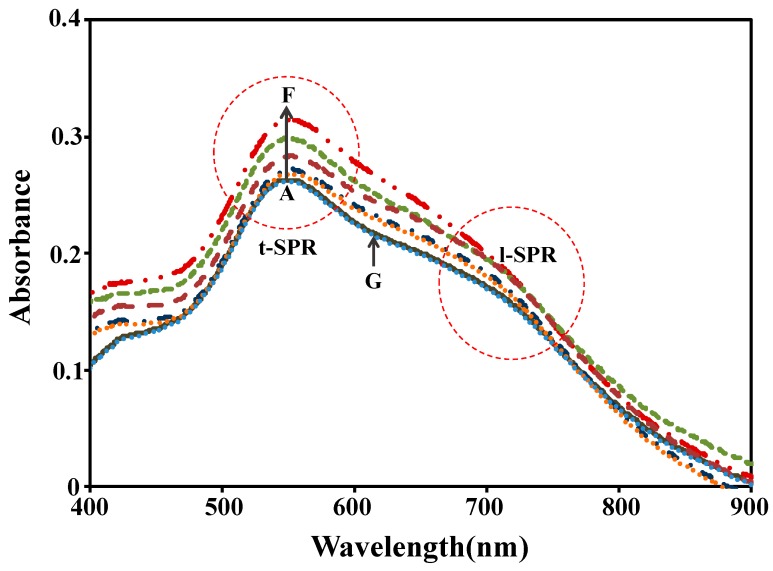
The plasmonic responses of Au nanoplates to the variation concentration of boric acid: (**A**) DI water, (**B**) 0.01 mM, (**C**) 0.1 mM, (**D**) 1 mM, (**E**) 10 mM, (**F**) 100 mM, and (**G**) 200 mM boric acid.

From the results, it was found that two changes in the spectra were recorded, namely, the change in the SPR peak position (see Figure 3) and its intensity. For the SPR intensity, we observed that the changes of intensity at t-SPR are almost the same as those at l-SPR for each boric acid concentration. 

**Figure 4:**
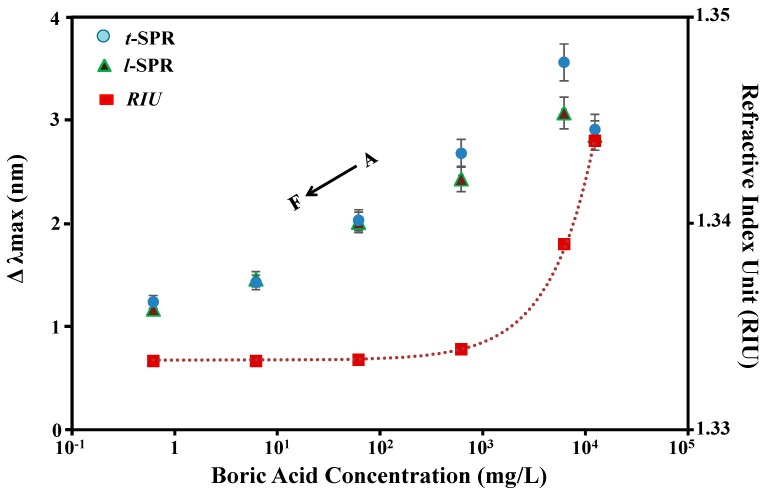
The plasmonic responses of transverse surface plasmon resonance (t-SPR) and longitudinal surface plasmon resonance (l-SPR) with the relationship between peak position and boric acid concentrations: (**A**) 200 mM, (**B**) 100 mM, (**C**) 10 mM, (**D**) 1 mM, (**E**) 0.1 mM, and (**F**) 0.01 mM boric acid. The dotted line represents the refractive index (RIU) of each boric acid concentration. * Refractive index for acid boric is 0.13339.

Similarly, it appears that the sensing responses of t-SPR are linearly correlated with the increasing of the boric acid concentration, where the linear correlation coefficient (*r*) is greater than 0.90. The (*r*) was generated by data samples from 0.01 mM to 100 mM. In addition, it was observed that the l-SPR response is not very fine because of uneven homogeneity of Au nanoplates, but this can be controlled and improved further. Then, it was found that when the sample of Au nanoplates was immersed into 150 mM and 200 mM of boric acid concentration, the intensity of the spectrum became attenuated. This can be explained as follows; when the solution becomes more concentrated, the boric acid will tend to silt in the bottom of the solution. This condition causes the properties of the solution to turn into particles. These particles will act as seeds that attract other seeds to thus clump together. Solubility properties of these materials will decrease and thus affect the sensitivity of the sensing being performed. In addition, these conditions may occur due to the weak resonance of particles caused by the close positioning of molecules, since the total energy from the light source used is constant. The increase of large molecules in the solution is caused by a high concentration of boric acid. Repeating sample testing for boric acid ≤100 mM has shown that similar output response can still be obtained. Thus, we confirmed that the sample was not damaged. In the case of peak position (see Figure 4), the change of t-SPR is slightly larger than l-SPR with almost the same (*r*). All changes in the SPR peak position and intensity are summarized in Table 1. The refractive index of pure boric acid was obtained from the manufacturer. In order to vary the concentration of boric acid, the solutions were diluted using DI water and the Refractive Index Unit (RIU) was calculated using the Arago-Biot equation.

**Figure 5:**
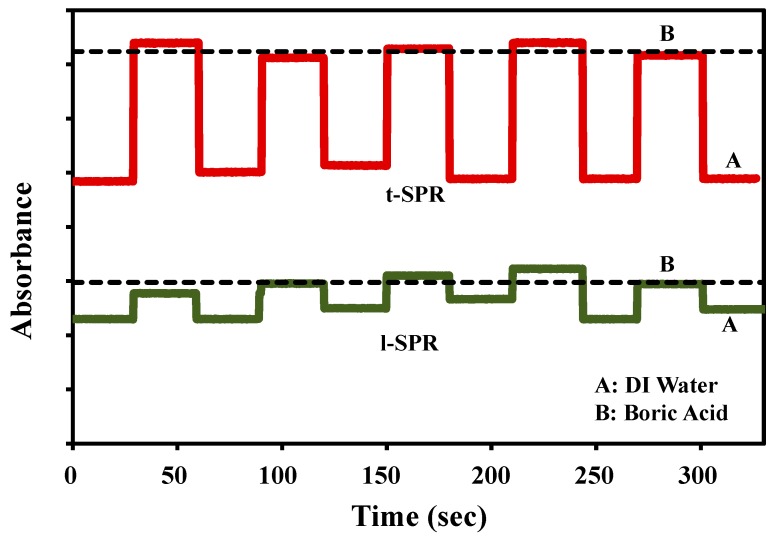
Plasmonic response of Au nanoplates towards boric acid and its corresponding response times with DI water as baseline. The dotted line represents the average peak intensity reading in the boric acid medium.

Then, a repeatability study of the sensing property of the Au nanoplates sample towards boric acid was carried out. Figure 5 shows the time responses of the Au nanoplates to the presence of 10 mM boric acid that was measured at both t-SPR and l-SPR. The reference used was DI water. In this study, the Au nanoplates sample was immersed in DI water and boric acid alternately every 30 s. The results indicate that the sensor gave fast response and recovery for at least five cycles. The stability of the response depends on the transmitted beam from the light source; hence, if the source is hot, the response will not be accurate.

## 4. Conclusions

The LSPR response of Au nanoplates to the presence of boric acid in water has been investigated. This study shows that the LSPR of Au nanoplates is sensitive towards the presence of boric acid in concentrations as low as 0.01 mM. The sensing parameters are based on the changes of the resonance peak position and intensity. The responses were found to increase linearly with the increase in the concentration of boric acid until it reached the saturation limit at 200 mM. Moreover, the repeatability study showed that the response of t-SPR is more stable than that of l-SPR. In addition, improving the quality of the sensing material could narrow the LSPR peak and further enhance the performance of the LSPR sensor.

## Figures and Tables

**Table 1:** Summary for all changes in the SPR peak position and intensity.

Parameter	∆Intensity (10^−3^)		∆Peak Position (nm)	
Concentration (mM)	t-SPR	l-SPR	t-SPR	l-SPR
0.01	6.67	6.25	1.24	1.17
0.1	10.00	9.75	1.43	1.46
1.0	15.75	15.30	2.03	2.01
10	20.00	20.00	2.68	2.41
100	31.25	31.33	3.56	3.07
200	22.33	29.00	2.91	2.85
